# Development of Interstitial Lung Disease Among Patients With Atrial Fibrillation Receiving Oral Anticoagulants in Taiwan

**DOI:** 10.1001/jamanetworkopen.2022.43307

**Published:** 2022-11-22

**Authors:** Yi-Hsin Chan, Tze-Fan Chao, Shao-Wei Chen, Hsin-Fu Lee, Wei-Min Chen, Pei-Ru Li, Yung-Hsin Yeh, Chi-Tai Kuo, Lai-Chu See, Gregory Y. H. Lip

**Affiliations:** 1 Cardiovascular Department, Chang Gung Memorial Hospital, Linkou, Taoyuan City, Taiwan; 2 College of Medicine, Chang Gung University, Taoyuan City, Taiwan; 3 Microscopy Core Laboratory, Chang Gung Memorial Hospital, Linkou, Taoyuan City, Taiwan; 4 Division of Cardiology, Department of Medicine, Taipei Veterans General Hospital, Taipei City, Taiwan; 5 Institute of Clinical Medicine, Cardiovascular Research Center, National Yang Ming Chiao Tung University, Taipei City, Taiwan; 6 Division of Thoracic and Cardiovascular Surgery, Department of Surgery, Chang Gung Memorial Hospital, Linkou Medical Center, Chang Gung University, Taoyuan City, Taiwan; 7 Graduate Institute of Clinical Medical Sciences, College of Medicine, Chang Gung University, Taoyuan City, Taiwan; 8 New Taipei City Municipal Tucheng Hospital, Chang Gung Memorial Hospital, Tucheng Branch, New Taipei City, Taiwan; 9 Department of Public Health, College of Medicine, Chang Gung University, Taoyuan City, Taiwan; 10 Biostatistics Core Laboratory, Molecular Medicine Research Center, Chang Gung University, Taoyuan City, Taiwan; 11 Division of Rheumatology, Allergy and Immunology, Department of Internal Medicine, Chang Gung Memorial Hospital, Linkou, Taoyuan City, Taiwan; 12 Liverpool Centre for Cardiovascular Science, University of Liverpool and Liverpool Heart and Chest Hospital, Liverpool, United Kingdom

## Abstract

**Importance:**

There are emerging concerns from case reports and pharmacovigilance analyses of a possible risk of interstitial lung disease (ILD) associated with the use of factor Xa (FXa) inhibitors.

**Objective:**

To evaluate the risk of incident ILD associated with the use of oral anticoagulants (OACs) in patients with nonvalvular atrial fibrillation (NVAF).

**Design, Setting, and Participants:**

This nationwide retrospective cohort study used data from the Taiwan National Health Insurance Research Database. Patients with NVAF without preexisting lung disease who received OACs from June 1, 2012, to December 31, 2017, were included. Propensity score stabilized weighting (PSSW) was used to balance covariates across the medication groups (FXa inhibitors, dabigatran, and warfarin, with warfarin as the reference). Patients were followed up from the drug index date until the onset of ILD, death, or end of the study (December 31, 2019), whichever occurred first. Data were analyzed from September 11, 2021, to August 3, 2022.

**Exposures:**

Patients with NVAF were treated with FXa inhibitors, dabigatran, or warfarin.

**Main Outcomes and Measures:**

New-onset idiopathic ILD.

**Results:**

Among the 106 044 patients (mean [SD] age, 73.4 [11.9] years; 59 995 men [56.6%]) included in the study, 64 555 (60.9%) received FXa inhibitors (apixban [n = 15 386], edoxaban [n = 12 413], and rivaroxaban [n = 36 756]), 22 501 (21.2%) received dabigatran, and 18 988 (17.9%) received warfarin at baseline. The FXa inhibitors were associated with a higher risk of incident ILD (0.29 vs 0.17 per 100 patient-years; hazard ratio, 1.54 [95% CI, 1.22-1.94]; *P* < .001), whereas dabigatran was associated with a nonsignificant difference in risk of incident ILD compared with warfarin (reference) after PSSW. The higher risk of incident ILD for FXa inhibitors vs warfarin was consistent with several high-risk subgroups.

**Conclusions and Relevance:**

Results of this study suggest that FXa inhibitors were associated with lung injury among patients with NVAF who were treated with OACs. Physicians should be vigilant in monitoring for any potential adverse lung outcomes associated with the use of these drugs.

## Key Points

**Question:**

Is the use of non–vitamin K antagonist oral anticoagulants assocciated with the development of interstitial lung disease (ILD)?

**Findings:**

In this cohort study of 106 044 patients with nonvalvular atrial fibrillation without preexisting lung disease, 64 555 patients received factor Xa (FXa) inhibitors, 22 501 received dabigatran, and 18 988 received warfarin at baseline. Use of FXa inhibitors was associated with a higher risk of incident ILD and use of dabigatran was associated with a nonsignificant difference in risk of incident ILD compared with warfarin after propensity score stabilized weighting; the higher risk of incident ILD associated with FXa inhibitors vs warfarin was observed in several high-risk subgroups.

**Meaning:**

Findings of the study suggest that physicians should be vigilant in monitoring for any potential adverse lung outcomes of FXa inhibitors.

## Teaser

This cohort study evaluates the risk of new-onset idiopathic interstitial lung disease among patients receiving factor Xa inhibitors, dabigatran, or warfarin.

## Introduction

In patients with atrial fibrillation (AF), the use of non–vitamin K antagonist oral anticoagulants (NOACs) has been found to be at least as effective in stroke prevention as the use of vitamin K antagonists and to be associated with a lower risk of major bleeding (except for rivaroxaban and dabigatran 150 mg) than the use of vitamin K antagonists (eg, warfarin) in clinical trials and clinical practice, even in several complex scenarios.^1,2,3,4,5^ Current international guidelines have recommended using NOACs as an effective, safe, and more convenient alternative to warfarin for stroke prevention among patients with nonvalvular AF (NVAF).^6,7^

Although various NOACs have been found to have lower bleeding risk profiles than warfarin,^1^ there are emerging concerns from case reports and pharmacovigilance analyses of a possible risk of interstitial lung disease (ILD) associated with the use of NOACs.^8^ Major clinical trials have reported that cough, dyspnea, and respiratory disorders are adverse events associated with common NOACs.^9,10,11^ For example, approximately 9% of patients treated with dabigatran reported the adverse event of dyspnea in the RE-LY (Randomized Evaluation of Long Term Anticoagulant Therapy With Dabigatran Etexilate) trial^9^; among patients treated with rivaroxaban, 5.34% reported dyspnea and 5.57% reported bronchitis in the ROCKET-AF (An Efficacy and Safety Study of Rivaroxaban With Warfarin for the Prevention of Stroke and Non-Central Nervous System Systemic Embolism in Patients With Non-Valvular Atrial Fibrillation) trial^10^; and 2.2% of patients treated with apixaban reported pneumonia in the ARISTOTLE (Apixaban for the Prevention of Stroke in Subjects With Atrial Fibrillation) trial.^11^ Cases of ILD associated with apixaban have also been reported, especially in Japanese study participants.^12^ However, to our knowledge, no large observational studies have comprehensively investigated postmarketing evidence of pulmonary toxic effects with apixaban until now. Moreover, because the previous study reported on ILD associated with apixaban only, no inferences can be drawn regarding the association between ILD and different NOACs. Therefore, further studies are necessary to address the safety of NOACs.

In this study, we aimed to evaluate the risk of incident ILD associated with the use of oral anticoagulants (OACs) in patients with NVAF. Specifically, we investigated whether factor Xa (FXa) inhibitor (apixaban, edoxaban, and rivaroxaban) or direct thrombin inhibitor (dabigatran) was associated with a risk of ILD compared with warfarin among patients treated with OACs who were enrolled in a large population-based nationwide cohort study in Taiwan.

## Methods

We performed a nationwide retrospective cohort study using the Taiwan National Health Insurance Research Database (NHIRD).^13^ The Chang Gung Medical Foundation Institutional Review Board approved this study and waived the informed consent requirement because of a consistent encrypting procedure used to deidentify the original identification number of each patient in the NHIRD. We followed the Strengthening the Reporting of Observational Studies in Epidemiology (STROBE) reporting guideline.

### Study Design

**Figure 1.**
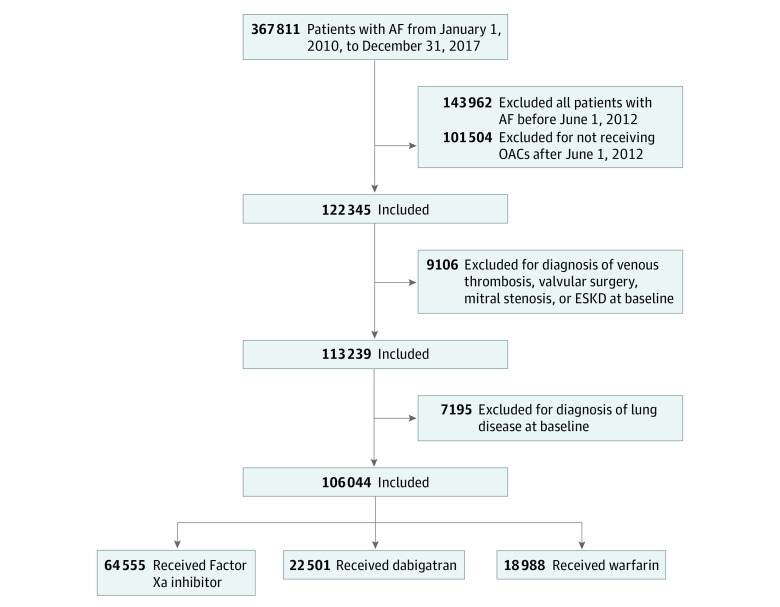
Study Flowchart Diagram A total of 106 044 patients with nonvalvular atrial fibrillation (AF) without preexisting lung disease receiving oral anticoagulants (OACs) were included. Among them were those treated with factor Xa inhibitor, dabigatran, or warfarin. ESKD indicates end-stage kidney disease.

The flowchart of study enrollment is shown in Figure 1 and eFigure 1 in the Supplement. In brief, we identified 367 811 patients who were diagnosed with AF (*International Classification of Diseases, Ninth Revision, Clinical Modification* [*ICD-9-CM*] code 427.31 or *International Statistical Classification of Diseases, Tenth Revision, Clinical Modification* [*ICD-10-CM*] code I48) between January 1, 2010, and December 31, 2017. Because OACs were reimbursed after June 2012 in Taiwan, we included records after June 2012. There were 113 239 patients with NVAF who were treated with OACs after we excluded the diagnosis of venous thromboembolism, valvular surgery, mitral stenosis, or end-stage kidney disease at baseline before the drug index date. After excluding 7195 patients with a diagnosis of any chronic lung disease before the drug index date, we enrolled 106 044 patients with NVAF without a previous diagnosis of chronic lung disease who were treated with NOACs (n = 87 056) or warfarin (n = 18 988).

The drug index date was defined as the first prescription date for NOACs or warfarin. The follow-up period was from the drug index date to the first occurrence of study outcome (ILD), death, or end of the study (December 31, 2019), whichever occurred first. This study, similar to most trials, was performed under an intention-to-treat principle, in which dropping out of anticoagulant therapy was not considered the study end point.

### Outcomes

The study outcome was new-onset idiopathic ILD (*ICD 9-CM* codes 515-516.9; *ICD-10-CM* codes J84-J84.9) with at least 1 principal inpatient or 2 outpatient diagnostic codes after the drug index date. The *ICD-9-CM* and *ICD-10-CM* codes indicating the diagnosis of idiopathic ILD were suggested by the American Thoracic Society in 2016 (eTable 1 in the Supplement).^14^ To explore potential residual confounding due to unmeasured confounders, we assessed the association between NOAC or warfarin initiation and the falsification outcomes of other lung diseases, including incident lung cancer or hospital admission due to influenza or asthma.^15^ We did not expect NOACs to be associated with either falsification outcomes; an association may point to residual confounding or information bias.

### Covariates

Baseline covariates were obtained from claims records, with the diagnoses, medications, or procedure codes, before the drug index date. Claims data on chronic medical conditions, cardiovascular disease risk factors, and bleeding events were collected for each participant within 12 months before the drug index date. The CHA_2_DS_2_-VASc (congestive heart failure, hypertension, age ≥75 years, diabetes, previous stroke or transient ischemic attack, vascular disease, age 65-74 years, female sex) score was used to calculate the risk of thromboembolism (score range: 0-9, with the highest score indicating the highest risk of stroke) in patients with NVAF who received OACs.^16^ The HAS-BLED (hypertension, abnormal kidney or liver function, stroke, bleeding history, labile international normalized ratio, age ≥65 years, antiplatelet drug or alcohol use) score was computed to estimate the risk of bleeding (score range: 0-9, with the highest score indicating the highest risk of bleeding) in patients with NVAF who were treated with OACs.^17^ Because there were no data to inform the labile international normalized ratio, this criterion was not included in the (modified) HAS-BLED calculation. The *ICD* codes used to identify the baseline covariates are summarized in eTable 2 in the Supplement. Only patients of Asian ethnicity were included in the study.

We also collected data on baseline medications for treatment of cardiovascular disease and other chronic medical conditions as well as data on potentially interacting medications that might alter NOAC or warfarin pharmacokinetics.^18^ The baseline medications were restricted to medications that were prescribed at least once within 3 months before the drug index date. The use of target therapy was defined as any prescription of mammalian target of rapamycin inhibitor, vascular endothelial growth factor inhibitor, 26S proteasome inhibitor, tyrosine kinase inhibitor, monoclonal antibodies, or cyclin-dependent kinase (CDK4 or CDK6) inhibitor for cancer treatment.

### Statistical Analysis

We used propensity score stabilized weighting (PSSW) to balance the differences in baseline characteristics across the medication groups (FXa inhibitors, dabigatran, and warfarin).^19,20^ All covariates in the Table were included in the generalized boosted models, except for CHA_2_DS_2_-VASc and HAS-BLED scores because these scores were already a combination of other covariates. One of the most valuable features of the generalized boosted models method is its iterative process with multiple regression trees to capture complex and nonlinear associations between medication groups and baseline characteristics. The generalized boosted models method then selects an intermediate iteration (or several trees) for the final model to minimize an imbalance in the baseline characteristics across the medication groups.^19,20^

**Table.** Baseline Characteristics of Patients With Nonvalvular AF Treated With FXa Inhibitor, Dabigatran, or Warfarin Before PSSW

Characteristic	Patients, No. (%)			ASMD	
	FXa inhibitor (n = 64 555)	Dabigatran (n = 22 501)	Warfarin (n = 18 988)	FXa inhibitor vs warfarin	Dabigatran vs warfarin
Age					
Mean (SD), y	74.7 (11.2)	73.3 (10.9)	69.4 (14.1)	0.4135	0.3105
<65	11 598 (18.0)	4550 (20.2)	7536 (39.7)	0.5009	0.4693
65-74	19 072 (29.5)	7322 (32.5)	3906 (20.6)	NA	NA
75-84	21 679 (33.6)	7441 (33.1)	4715 (24.8)	NA	NA
≥85	12 206 (18.9)	3188 (14.2)	2831 (14.9)	NA	NA
Sex					
Male	35 646 (55.2)	13 589 (60.4)	10 760 (56.7)	0.0292	0.0757
Female	28 909 (44.8)	8912 (39.6)	8228 (43.3)	NA	NA
CHA_2_DS_2_-VASc score, mean (SD)	3.3 (1.7)	3.2 (1.6)	2.6 (1.9)	0.3748	0.3140
HAS-BLED score, mean (SD)	2.6 (1.2)	2.5 (1.1)	2.1 (1.3)	0.3975	0.3437
Hypertension	34 387 (53.3)	10 931 (48.6)	7904 (41.6)	0.2347	0.1401
Diabetes	23 317 (36.1)	7786 (34.6)	5702 (30.0)	0.1297	0.0979
Dyslipidemia	29 329 (45.4)	9225 (41.0)	6411 (33.8)	0.2403	0.1500
Chronic liver disease	5569 (8.6)	1767 (7.9)	1485 (7.8)	0.0293	0.0012
CKD	11 238 (17.4)	2647 (11.8)	2600 (13.7)	0.1027	0.0579
Gout	10 235 (15.9)	3110 (13.8)	2590 (13.6)	0.0625	0.0053
CHF	5749 (8.9)	1644 (7.31)	1769 (9.3)	0.0143	0.0729
Chronic IHD	7289 (11.3)	1993 (8.9)	1650 (8.7)	0.0868	0.0059
Stroke	11 900 (18.4)	5382 (23.9)	2459 (13.0)	0.1512	0.2857
Cancer	6822 (10.6)	1920 (8.5)	1665 (8.8)	0.0609	0.0084
RA	223 (0.4)	74 (0.3)	49 (0.3)	0.0159	0.0131
PCI	4694 (7.3)	1161 (5.2)	955 (5.0)	0.0934	0.0059
CABG	354 (0.6)	59 (0.3)	210 (1.1)	0.0616	0.1025
History of bleeding	1029 (1.6)	305 (1.4)	305 (1.6)	0.0010	0.0208
Use of NSAIDs	15 955 (24.7)	5332 (23.7)	4866 (25.6)	0.0210	0.0448
Use of PPI	7987 (12.4)	2069 (9.2)	2551 (13.4)	0.0317	0.1341
Use of H_2_RB	20 309 (31.5)	7140 (31.7)	6132 (32.3)	0.0179	0.0120
Use of ACEI, ARB subtype II	38 543 (59.7)	13 320 (59.2)	10 539 (55.5)	0.0851	0.0747
Use of β-blocker	39 399 (61.0)	13 000 (57.8)	11 739 (61.8)	0.0163	0.0826
Use of verapamil or diltiazem	14 730 (22.8)	4744 (21.1)	4685 (24.7)	0.0436	0.0855
Use of statin	23 061 (35.7)	7751 (34.5)	4835 (25.5)	0.2240	0.1971
Use of APT	34 563 (53.5)	12 156 (54.0)	10 455 (55.1)	0.0305	0.0208
Use of amiodarone	19 787 (30.7)	5567 (24.7)	8065 (42.5)	0.2474	0.3822
Use of dronedarone	2659 (4.1)	353 (1.6)	322 (1.7)	0.1446	0.0100
Use of chemotherapy	988 (1.5)	256 (1.1)	263 (1.4)	0.0121	0.0222
Use of target therapy	999 (1.6)	215 (1.0)	141 (0.7)	0.0757	0.0232
Use of methotrexate	183 (0.3)	56 (0.3)	51 (0.3)	0.0028	0.0039
Use of anti-TNF agent	38 (0.1)	15 (0.1)	11 (0.1)	0.0004	0.0035
Use of corticosteroid	1713 (2.7)	464 (2.1)	583 (3.1)	0.0250	0.0638
Use of quinidine	70 (0.1)	23 (0.1)	49 (0.3)	0.0350	0.0368
Use of rifampicin	194 (0.3)	56 (0.3)	74 (0.4)	0.0152	0.0250
Use of macrolides	1452 (2.3)	387 (1.7)	504 (2.7)	0.0262	0.0639
Use of antifungal agent	571 (0.9)	113 (0.5)	261 (1.4)	0.0464	0.0906

Abbreviations: ACEI, angiotensin-converting enzyme inhibitor; AF, atrial fibrillation; APT, antiplatelet therapy; ARB, angiotensin receptor blocker; ASMD, absolute standardized mean difference; CABG, coronary artery bypass graft; CHA_2_DS_2_-VASc, congestive heart failure, hypertension, age 75 years or older, diabetes, previous stroke or transient ischemic attack, vascular disease, aged 65 to 74 years, female sex; CHF, congestive heart failure; CKD, chronic kidney disease; FXa, factor Xa; H_2_RB, histamine-2 receptor blocker; HAS-BLED, hypertension, abnormal kidney or liver function, stroke history, bleeding history, labile international normalized ratio, age 65 years or older, antiplatelet drug or alcohol use; IHD, ischemic heart disease; NA, not applicable; NSAID, nonsteroidal anti-inflammatory drug; PCI, percutaneous coronary intervention; PPI, proton pump inhibitor; PSSW, propensity score stabilized weighting; RA, rheumatoid arthritis; TNF, tumor necrosis factor.

The balance of potential confounders at baseline (drug index date) was assessed using the absolute standardized mean difference (ASMD), and ASMD of 0.1 or less indicated an insignificant difference in potential confounders across the medication groups. The incidence rates were computed by dividing the total number of study outcomes during the follow-up period by person-years at risk. The risk of study outcomes for NOACs vs warfarin (reference) was obtained through survival analysis using the Kaplan-Meier method and Cox proportional hazards regression model.

Subgroup analysis was performed to test whether the hazard ratios (HRs) in a specific subgroup (NOACs vs warfarin) were similar to the HRs of the overall group. Sensitivity analysis was also performed to examine the robustness of the main findings. Given that patients who live longer have more time to develop ILD, we used the cause-specific hazard model, which accounted for death as a competing risk, to estimate the subdistribution HR.^21^ We performed PSSW for the subgroup analysis and sensitivity analysis to ensure the covariates’ balance across the medication groups.

A 2-sided *P* < .05 indicated statistical significance. All statistical analyses were performed with SAS, version 9.4 (SAS Institute Inc). Data were analyzed from September 11, 2021, to August 3, 2022.

## Results

Of the 106 044 patients with NVAF (mean [SD] age, 73.4 [11.9] years; 59 995 men [56.6%] and 46 049 women [43.4%]) included, 64 555 (60.9%) received FXa inhibitors, 22 501 (21.2%) received dabigatran, and 18 988 (17.9%) received warfarin (Figure 1). Before PSSW, both the FXa inhibitor and dabigatran groups were older; had higher CHA_2_DS_2_-VASc and HAS-BLED scores; and had several comorbidities, including hypertension, dyslipidemia, and stroke, compared with the warfarin group (ASMD >0.1) (Table). After PSSW, all medication groups were well balanced in all characteristics (all ASMD <0.1) (eTable 3 in the Supplement).

### Primary Outcome

**Figure 2.**
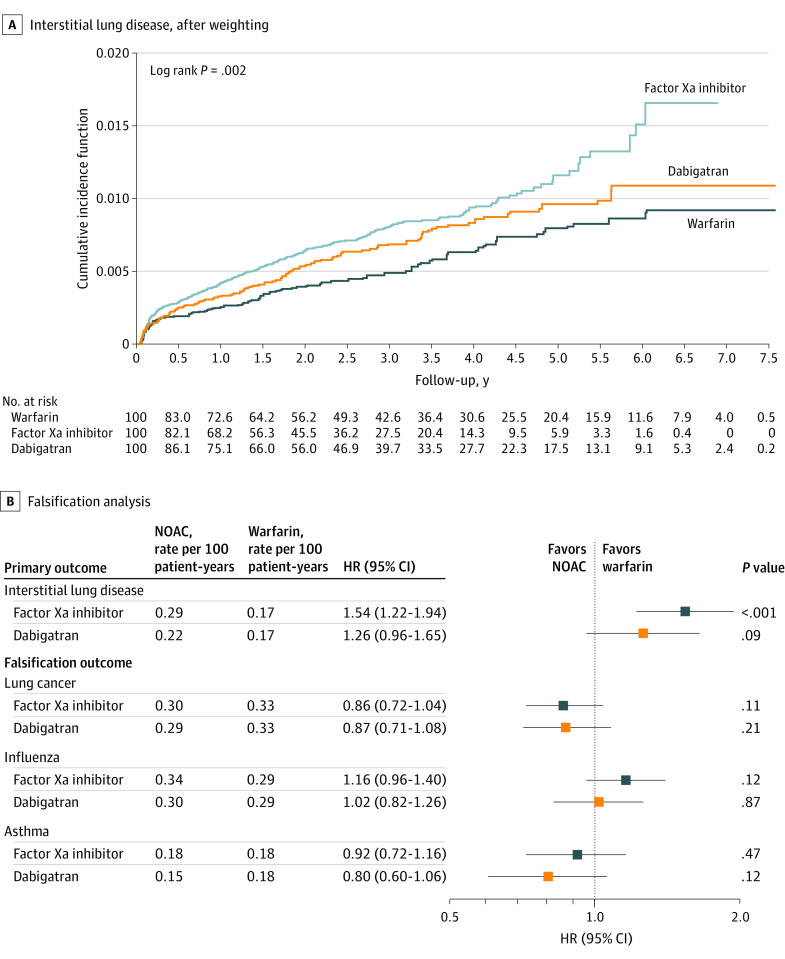
Cumulative Incidence Curves and Forest Plot of Hazard Ratio (HR) of Interstitial Lung Disease (ILD) for Patients Treated With Factor Xa (FXa) Inhibitor, Dabigatran, or Warfarin After Propensity Score Stabilized Weighting A, The FXa inhibitor group had a higher risk of incident ILD, whereas the dabigatran group had a comparable risk of incident ILD vs the warfarin group after weighting. B, The falsification analysis indicated that neither FXa inhibitor nor dabigatran was associated with a decreased or increased risk of irrelevant events compared with warfarin after weighting. NOAC indicates non–vitamin K antagonist oral anticoagulant.

Cumulative incidence curves of ILD among the medication groups after PSSW are shown in Figure 2A. The FXa inhibitor group had a higher risk of incident ILD (0.29 vs 0.17 per 100 patient-years; HR, 1.54 [95% CI, 1.22-1.94]; *P* < .001), whereas the dabigatran group had a nonsignificant difference in risk of incident ILD compared with the warfarin group after PSSW. Use of FXa inhibitor was associated with an absolute risk increase of 0.12 (95% CI, 0.08-0.17) per 100 patient-years and use of dabigatran was associated with an absolute risk increase of 0.05 (95% CI, −0.001 to 0.10) per 100 patient-years among patients with NVAF after PSSW. We also examined the risk of thromboembolism and major bleeding among patients with NVAF treated with FXa inhibitor, dabigatran, or warfarin during the follow-up period. Use of FXa inhibitor was associated with an absolute risk decrease of 0.78 (95% CI, 0.63-0.94) per 100 patient-years for ischemic stroke or systemic embolism and 0.78 (95% CI, 0.63-0.93) per 100 patient-years for major bleeding compared with use of warfarin (eTable 4 in the Supplement). For those who received dabigatran, use was associated with an absolute risk decrease of 0.64 (95% CI, 0.46-0.82) per 100 patient-years for ischemic stroke or systemic embolism and 1.01 (95% CI, 0.84-1.17) per 100 patient-years for major bleeding (eTable 4 in the Supplement).

Among patients who were diagnosed with ILD during the follow-up period, antifibrotic agents (pirfenidone or nintedanib) were received by 9.18 per 100 patients in the FXa inhibitor group, 5.56 per 100 patients in the dabigatran group, and 3.03 per 100 patients in the warfarin group. Immunosuppressants (corticosteroid, tacrolimus, cyclosporine, rituximab, cyclophosphamide, mycophenolate, or azathioprine) were provided to 68.73 per 100 patients in the FXa inhibitor group, 68.25 per 100 patients in the dabigatran group, and 74.75 per 100 patients in the warfarin group.

Patients who were diagnosed with ILD during the follow-up period and treated with FXa inhibitors had a higher risk of ILD requiring consequent antifibrotic agents than those who were treated with warfarin (odds ratio [OR], 3.01; 95% CI, 0.87-10.46; *P* = .03). There were no significant differences in the risk of ILD requiring consequent immunosuppressants for the 3 medication groups with ILD diagnosis. A falsification analysis indicated that neither FXa inhibitor nor dabigatran was associated with a decreased or increased risk of irrelevant events compared with warfarin after PSSW (Figure 2B).

### Subgroup Analysis

Subgroup analyses were performed to ascertain whether different FXa inhibitors were associated with a higher risk of incident ILD than warfarin. For the FXa inhibitor group, 23.8% (n = 15 386 of 64 555) of patients were treated with apixaban, 19.2% (n = 12 413 of 64 555) were treated with edoxaban, and 56.9% (n = 36 756 of 64 555) were treated with rivaroxaban. All 3 FXa inhibitors were associated with a significantly higher risk of incident ILD (apixaban: 0.35 vs 0.17 per 100 patient-years [HR, 1.72; 95% CI, 1.27-2.31]; edoxaban: 0.37 vs 0.17 per 100 patient-years [HR, 1.60; 95% CI, 1.12-2.27]; rivaroxaban: 0.27 vs 0.17 per 100 patient-years [HR, 1.48; 95% CI, 1.16-1.88]; *P* = .75) compared with warfarin after PSSW (eFigure 2 in the Supplement).

Subgroup analysis showed that a higher risk of incident ILD for the FXa inhibitor group vs the warfarin group was consistent regardless of age; sex; CHA_2_DS_2_-VASc score; HAS-BLED score; use of angiotensin system inhibitor, amiodarone, statin, or β-blocker; or risk of stroke or major bleeding (for example, age <75 years: 0.24 vs 0.12 per 100 patient-years [HR, 1.72; 95% CI, 1.24-2.41]; age ≥75 years: 0.37 vs 0.22 per 100 patient-years [HR, 1.58; 95% CI, 1.12-2.23]; *P* = .73) (eFigure 2 in the Supplement). Conversely, the use of dabigatran was not associated with a risk of incident ILD for all subgroups compared with warfarin use (eFigure 3 in the Supplement).

Amiodarone, frequently coprescribed with NOACs in patients with AF, was prescribed in 30.7% of patients treated with FXa inhibitors, 24.7% of patients treated with dabigatran, and 42.5% of patients treated with warfarin (Table). Those in the FXa inhibitor group received a median (IQR) of 49 (0-1136) days of baseline amiodarone before the drug index date, whereas the median (IQR) was 46 (0-939) days for those in the dabigatran group and 3 (0-352) days for those in the warfarin group. Use of amiodarone was associated with a higher risk of incident ILD in patients who were treated with FXa inhibitors (0.38 vs 0.26 per 100 patient-years; HR, 1.41 [95% CI, 1.15-1.73]; *P* < .001), dabigatran (0.31 vs 0.18 per 100 patient-years; HR, 1.62 [95% CI, 1.12-2.35]; *P* = .01), or warfarin (0.28 vs 0.13 per 100 patient-years; HR, 1.97 [95% CI, 1.32-2.95]; *P* < .001). Patients who received both amiodarone and FXa inhibitors had the highest risk of incident ILD, whereas those treated with warfarin but without amiodarone had the lowest risk of incident ILD (0.38 vs 0.13 per 100 patient-years; HR, 2.69 [95% CI, 1.88-3.85]; *P* < .001) (eFigure 4 in the Supplement).

### Sensitivity Analysis

We performed several sensitivity analyses to examine the robustness of the present results. First, after considering death as a competing risk, use of FXa inhibitors was still associated with a higher risk of ILD than warfarin use. However, the dabigatran group had a significantly higher risk of ILD than the warfarin group (HR, 1.36; 95% CI, 1.04-1.78; *P* = .03), a different finding from the main finding, which did not account for the competing risk of death. Second, we restricted the ILD that required 1 or more claims within 30 to 365 days of the first diagnosis of ILD^22^ and observed results similar to those for the ILD without this requirement. Third, we restricted patients who were treated with NOAC without previous warfarin exposure for at least 180 days before NOAC initiation. The number of patients without previous warfarin exposure was 61 713 (95.6%) for the FXa inhibitor group and 21 035 (93.5%) for the dabigatran group. The baseline characteristics before and after PSSW are summarized in eTables 5 and 6 in the Supplement. After PSSW, the 3 medication groups were well balanced in all characteristics (all ASMD<0.1) (eTable 6 in the Supplement). Significant difference in ILD incident between the FXa inhibitor vs warfarin groups remained (0.30 vs 0.17 per 100 patient-years; HR, 1.56 [95% CI, 1.23-1.97]; *P* < .001).

**Figure 3.**
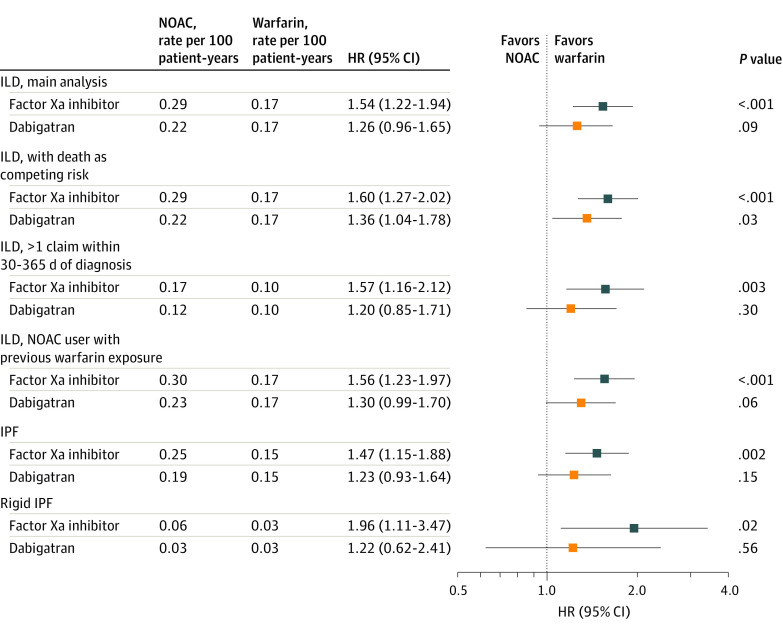
Sensitivity Analyses for Incident Interstitial Lung Disease (ILD) for the Patients Treated With Factor Xa (FXa) Inhibitor, Dabigatran, or Warfarin After Propensity Score Stabilized Weighting The use of FXa inhibitor was still associated with a higher risk of new-onset ILD vs warfarin after weighting, with death as a competing risk factor, consistent with the main analyses. In restricting the ILD requiring 1 or more claims within 30 to 365 days of the first diagnosis of ILD, idiopathic pulmonary fibrosis (IPF) or rigid IPF, or use of non–vitamin K antagonist oral anticoagulant (NOAC) without previous warfarin exposure for at least 180 days before NOAC initiation, the sensitivity analyses results remained consistent with the main findings. HR indicates hazard ratio.

Fourth, we focused on the idiopathic pulmonary fibrosis (IPF), a subcategory of ILD (*International Classification of Diseases, Ninth Revision* [*ICD-9*] codes 515, 516.30, 516.31, and 516.32 or *International Statistical Classification of Diseases and Related Health Problems, Tenth Revision* [*ICD-10*] codes J84.10, J84.89V, J84.111, J84.112, and J84.113). Idiopathic pulmonary fibrosis often requires detection by computed tomography, making misclassification unlikely. The incident rates of IPF were similar to the incident rates of ILD, suggesting that most ILD cases were also IPF cases. Again, we found a significantly higher ILD rate in the FXa inhibitor group than in the warfarin group. Fifth, when we further restricted IPF to a rigid IPF (*ICD-9* code 516.31 or *ICD-10* code J84.112), the incident rates of rigid IPF were much lower than the incident rates of ILD. The HR results of rigid IPF were similar to those of IPF, except with a larger 95% CI of the HR due to the small number of rigid IPF (0.06 vs 0.03 per 100 patient-years; HR, 1.96 [95% CI, 1.11-3.47]; *P* = .02) (Figure 3).

## Discussion

To our knowledge, this cohort study was the first population-based investigation of the risk of incident ILD in patients with NVAF who received NOAC vs warfarin treatment. The results showed that the 3 FXa inhibitors (apixaban, edoxaban, and rivaroxaban) were all associated with a higher risk of incident ILD compared with warfarin among patients with NVAF without preexisting lung disease. The outcomes were consistent across several high-risk subgroups. Conversely, dabigatran was not associated with a higher risk of incident ILD compared with warfarin. Use of amiodarone was associated with a higher risk of incident ILD in patients who were treated with FXa inhibitor, dabigatran, or warfarin.

Use of NOACs appears to have increased steadily over time worldwide.^23^ Since their approval in 2009, evidence has started to accumulate on the postmarketing surveillance of rare and non–hemorrhage-related adverse outcomes associated with NOACs, including liver toxic effects, thrombocytopenia, allergy, and hypersensitivity reactions. Concerns have also been raised on whether different NOACs can be considered a homogeneous pharmacological class, especially from a safety perspective.^24^

Major clinical trials have shown that dyspnea, respiratory distress, and pneumonia are adverse events associated with the use of NOACs^9,10,11^; therefore, we hypothesized that NOACs might be associated with an increased risk of incident ILD. With the ever-increasing number of NOACs worldwide, case series studies of incident ILD have been conducted, especially in Japanese populations.^12,25^ However, no large studies have comprehensively evaluated the risk of pulmonary adverse events among patients who were treated with NOACs in clinical practice.

Raschi et al^8^ summarized a hypothesis-generating study that found an association between ILD and NOACs, especially FXa inhibitors, with disproportionality signals between specific oral anticoagulants. The authors used the US Food and Drug Administration Adverse Event Reporting System to investigate the reporting of ILD associated with NOACs from 2004 to 2019. A total of 962 reports of ILD from 24 720 patients who received NOACs were identified, with 60% of patients being from Asia, 34% in female patients, and 87% in patients older than 65 years.^8^ Apixaban, edoxaban, and rivaroxaban consistently emerged with higher-than-expected reporting of ILD, whereas dabigatran did not.^8^ Reports from a non-Japanese population confirmed that apixaban and edoxaban were associated with a higher risk of ILD, whereas dabigatran and rivaroxaban were not associated with a higher risk.^8^

Raschi et al^8^ found disproportionality for edoxaban in the main analysis (reporting OR, 8.04; 95% CI, 6.47-9.79), which was confirmed by sensitivity analyses.^8,26^ Further analysis indicated that edoxaban was associated with a substantially higher disproportionality for ILD reporting compared with other NOACs either in the overall population or the non-Japanese population.^27^ Conversely, the pivotal randomized clinical trials comparing the effectiveness and safety of edoxaban (n = 18 212) vs warfarin (n = 11 185) in patients with NVAF (ENGAGE AF-TIMI 48 trial [Global Study to Assess the Safety and Effectiveness of Edoxaban (DU-176b) vs Standard Practice of Dosing With Warfarin in Patients With Atrial Fibrillation]) and in patients with venous thromboembolism (Hokusai-VTE trial [Comparative Investigation of Low Molecular Weight (LMW) Heparin/Edoxaban Tosylate (DU176b) Versus (LMW) Heparin/Warfarin in the Treatment of Symptomatic Deep-Vein Blood Clots and/or Lung Blood Clots]) were independently reviewed by a pulmonary expert for 160 suspected cases of incident ILD, but the analysis showed no clear signal of drug-induced ILD identified in these 2 phase 3 global edoxaban trials.^26,28,29^ In addition, the global and prospective ETNA-AF (Edoxaban Treatment in Routine Clinical Practice for Patients With Non Valvular Atrial Fibrillation) and ETNA-VTE (Edoxaban Treatment in Routine Clinical Practice in Patients With Venous Thromboembolism in Europe) registries reported a rare incidence of 5 of 11 190 patients (0.04%) with ILD who were treated with edoxaban after 2 years of follow-up.^26^

The reason for the higher risk of incidental ILD associated with all FXa inhibitors compared with warfarin cannot be conclusively ascertained given the observational nature of the present study. The findings were consistent with the results reported by Raschi et al^8^ that apixaban and edoxaban were associated with statistically significant disproportionality signals of ILD. One could hypothesize that confounding variables in the choice of FXa inhibitors rather than warfarin were responsible for the worse outcomes noted, even though the 2 study groups were well balanced in all characteristics after PSSW. An important comorbidity we were unable to account for, which might have prompted the use of warfarin, was impaired kidney function. However, it is not reasonable to assume that patients with impaired kidney function (treated with warfarin) could have a lower risk of incidental ILD than those with better kidney function (treated with FXa inhibitors). Furthermore, we cannot explain why dabigatran was the only NOAC that was not associated with a higher risk of incidental ILD compared with warfarin despite no clear demographic differences noted between the FXa inhibitors and dabigatran groups.

A few small clinical studies have reported on the safety of dabigatran use in patients with ILD.^30,31^ Warfarin can also be a factor in decreased inflammation and thrombin generation by reducing plasma factor VII, an important thrombin precursor in the coagulation cascade. In addition, factor VII leaks from damaged vessels into the lung interstitium in ILD would induce interleukin 6 production and enhance migration of resident fibroblasts, which are associated with chronic inflammation and thus contribute to fibrotic disease progression. Targeting factor XII–induced fibroblastic processes in pulmonary fibrosis may therefore ameliorate the progression of ILD.^32^ Furthermore, increased local concentration in lung interstitium and alveolar hemorrhage was suggestive of (and manifested as) ILD with FXa inhibitors, which have higher drug-drug interaction (eg, with amiodarone, which was frequently coprescribed with NOACs in patients with AF and was known to be associated with a higher risk of ILD^33^) and bleeding risk compared with dabigatran.^18^ The possible modifying factor of ethnicity (eg, Asian in this study) needs further evaluation.

Pulmonary toxic effects, including interstitial pneumonitis, eosinophilic pneumonia, organizing pneumonia, diffuse alveolar hemorrhage, and acute respiratory distress syndrome, are among the most severe adverse outcomes of amiodarone treatment.^34,35^ Interstitial pneumonitis is the most common presentation of amiodarone-induced pulmonary disease, especially in patients for whom the dose of amiodarone exceeds 400 mg/d.^36^ The underlying mechanisms in amiodarone-induced interstitial pneumonitis include a secondary to direct toxic injury to lung cells and an indirect immunologic reaction.^37^ Amiodarone is frequently coprescribed with NOACs for rhythm or rate control in patients with AF.^38^ Concurrent use of amiodarone and NOACs has been associated with increased risk of major bleeding compared with NOACs alone,^39^ which is possibly mediated by an increase of plasma NOAC levels with pharmacokinetic interaction (P-glycoprotein [P-gp] inhibition) with amiodarone.^18^ The present study found that the use of amiodarone was associated with a higher risk of incident ILD in patients treated with either FXa inhibitor, dabigatran, or warfarin. Patients who were treated with both amiodarone and FXa inhibitors had the highest risk of incident ILD. In addition, FXa inhibitors (especially rivaroxaban and apixaban) are metabolized mainly by the liver, mediated mainly via the cytochrome P (CYP) 3A4-type CYP450-dependent elimination.^18^ CYP3A4 inhibition or induction of a specific drug may affect plasma concentrations of NOACs and the concomitant drug itself. However, it is unclear whether coprescribing of an FXa inhibitor with amiodarone and other specific drugs (eg, several cardiovascular, anti-inflammatory, antimicrobial, biological, or antitumor agents, which may induce ILD^33^) was associated with increased plasma levels of FXa inhibitor, amiodarone, and other specific drugs, leading to the vulnerability in the development of ILD during the long-term follow-up period. The possible modifying factor or pharmacokinetic interaction of NOAC in these drugs requires elucidation.

The objectives and results of the present study did not mean to suggest that patients who were already being treated with NOACs (especially FXa inhibitors) change back to warfarin. The absolute difference in rates of ILD between the FXa inhibitors and warfarin was small (0.12 per 100 patient-years) and was much lower than the absolute reduction of in the incidence of thromboembolism (0.78 per 100 patient-years) and major bleeding (0.78 per 100 patient-years) between the FXa inhibitor and warfarin groups (eTable 4 in the Supplement).^1,2,3,4,5^ The results did not address the discontinuation of NOACs when ILD developed after NOAC initiation but suggested the need for close monitoring of lung function and clinical respiratory symptoms or signs in patients with NVAF during their background NOAC treatment. Moreover, patient adherence to treatment should be confirmed in those receiving NOACs who are concerned about the potential drug-drug interaction between NOACs and amiodarone as well as other drugs that are strong inhibitors or inducers of both P-gp and/or CYP3A. Any adverse events should be monitored (outside the bleeding or thromboembolic events) in patients with NVAF being treated with NOACs.

### Limitations

This study has several limitations. First, it relied solely on claims data without access to clinical data on ILD, which may lead to a biased estimate of the incidence of ILD. The incidence of ILD in Taiwan that we reported was higher than the crude incidence of ILD in the US reported by Olson et al.^22^ The broad diagnosis of idiopathic ILD suggested by the American Thoracic Society^14^ was different from the diagnosis codes indicating ILD that were adopted by Olson et al.^22^ In addition, the incidence of ILD increased with advancing patient age. Considering the routine prescription of OACs in older adults with AF (mean [SD] age, 73.4 [11.9] years in this study), it is possible that the overall patient population with AF treated with OACs had a higher risk of incident ILD. Furthermore, difference in geography, ethnicity, or methods may have resulted in diversity in the prevalence and incidence of ILD. Additional studies are needed to refine and/or replicate these findings and to validate the estimates. Second, different NOACs or warfarin had varying degrees of liver or kidney elimination; thus, the decision regarding the use of specific NOACs or warfarin may be guided by each patient’s liver or kidney function. However, laboratory data were lacking in the NHIRD.

Third, potential misclassification and miscoding of the underlying covariates and outcomes registered by each physician’s choice of treatment constituted an additional limitation of the present study. Fourth, we used the PSSW method to balance the baseline covariate for the medication groups. Before PSSW, patients in the FXa inhibitor and dabigatran groups were older, had higher CHA_2_DS_2_-VASc and HAS-BLED scores, and had several comorbidities compared with the warfarin group. After PSSW, all groups were well balanced in all characteristics. Although the PSSW allowed the balance of comorbidities among the groups, residual confounding by unmeasured variables and selective prescribing behavior could not be excluded. The channeling bias may still affect the study, which is likely to reflect a selective prescribing behavior. Fifth, smoking and exposure to occupational and environmental toxins were important risk factors in making patients more susceptible to developing ILD.^40^ However, we were unable to capture information on smoking or exposure to environmental toxins due to the limitations of the retrospective claims database.

Sixth, ILD can be attributed to use of several antiarrhythmic drugs, antibiotics, chemotherapeutic agents, and immunosuppressive agents.^33^ Although we selected an extensive number of baseline medications for the PSSW method and a close balance for those medications was achieved after PSSW, residual confounding by unmeasured drugs cannot be excluded. Seventh, we enrolled only Asian patients, and whether the results can be directly extrapolated to Western populations remains unclear.

## Conclusions

In this cohort study, FXa inhibitors appeared to be associated with lung injury among patients with NVAF who were treated with OACs. Physicians should be vigilant in monitoring for any potential adverse outcomes for the lungs associated with use of these drugs.

## Notes

Supplement. **eTable 1.** International Classification of Disease (9th and 10th Edition) Clinical Modification (ICD 9-CM and ICD 10-CM) Codes Used to Define the Idiopathic Interstitial Lung Disease (ILD) in the Study Cohort **eTable 2.** International Classification of Disease (9th and 10th Edition) Clinical Modification (ICD 9-CM and ICD 10-CM) Codes Used to Define the Co-Morbidities and Clinical Outcomes in the Study Cohort **eTable 3.** Baseline Characteristics of Patients With Non-Valvular AF Treated With Factor-Xa Inhibitor, Dabigatran, or Warfarin After PSSW **eTable 4.** The Incidence and Absolute Rate Difference of Interstitial Lung Disease (ILD), Thromboembolism, and Major Bleeding Among Patients With Non-Valvular AF Treated With Factor-Xa Inhibitor, Dabigatran, or Warfarin After PSSW **eTable 5.** Baseline Characteristics of Non-Valvular AF Patients Treated With Factor-Xa Inhibitor, Dabigatran, or Warfarin Before PSSW: NOAC Without Prior Warfarin Exposure (At Least 180 Days Wash-Out Period) **eTable 6.** Baseline Characteristics of Non-Valvular AF Patients Treated With Factor-Xa Inhibitor, Dabigatran, or Warfarin After PSSW: NOAC Without Prior Warfarin Exposure (At Least 180 Days Wash-Out Period) **eFigure 1.** Graphical Depiction of Longitudinal Study Designs **eFigure 2.** Subgroup Analysis of Hazard Ratio (HR) for Interstitial Lung Disease (ILD) for Patients Non-Valvular Atrial Fibrillation (AF) Treated With Factor-Xa Inhibitor and Warfarin After Propensity Score Stabilized Weights (PSSW) **eFigure 3.** Subgroup Analysis of HR for ILD for Non-Valvular AF Patients Treated With Dabigatran and Warfarin After PSSW **eFigure 4.** The Impact of Amiodarone on the Risk of ILD in Non-Valvular AF Patients Treated With Factor-Xa Inhibitor, Dabigatran, or Warfarin After PSSW
