# Supplementary material for: Development of Interstitial Lung Disease Among Patients With Atrial Fibrillation Receiving Oral Anticoagulants in Taiwan
Source: JAMA Netw Open. 2022 Nov 22;5(11):e2243307. doi: 10.1001/jamanetworkopen.2022.43307 (PMC9682427; doi:10.1001/jamanetworkopen.2022.43307)
Supplement: Supplement. — eTable 1. International Classification of Disease (9th and 10th Edition) Clinical Modification (ICD 9-CM and ICD 10-CM) Codes Used to Define the Idiopathic Interstitial Lung Disease (ILD) in the Study Cohort eTable 2. International Classification of Disease (9th and 10th Edition) Clinical Modification (ICD 9-CM and ICD 10-CM) Codes Used to Define the Co-Morbidities and Clinical Outcomes in the Study Cohort eTable 3. Baseline Characteristics of Patients With Non-Valvular AF Treated With Factor-Xa Inhibitor, Dabigatran, or Warfarin After PSSW eTable 4. The Incidence and Absolute Rate Difference of Interstitial Lung Disease (ILD), Thromboembolism, and Major Bleeding Among Patients With Non-Valvular AF Treated With Factor-Xa Inhibitor, Dabigatran, or Warfarin After PSSW eTable 5. Baseline Characteristics of Non-Valvular AF Patients Treated With Factor-Xa Inhibitor, Dabigatran, or Warfarin Before PSSW: NOAC Without Prior Warfarin Exposure (At Least 180 Days Wash-Out Period) eTable 6. Baseline Characteristics of Non-Valvular AF Patients Treated With Factor-Xa Inhibitor, Dabigatran, or Warfarin After PSSW: NOAC Without Prior Warfarin Exposure (At Least 180 Days Wash-Out Period) eFigure 1. Graphical Depiction of Longitudinal Study Designs eFigure 2. Subgroup Analysis of Hazard Ratio (HR) for Interstitial Lung Disease (ILD) for Patients Non-Valvular Atrial Fibrillation (AF) Treated With Factor-Xa Inhibitor and Warfarin After Propensity Score Stabilized Weights (PSSW) eFigure 3. Subgroup Analysis of HR for ILD for Non-Valvular AF Patients Treated With Dabigatran and Warfarin After PSSW eFigure 4. The Impact of Amiodarone on the Risk of ILD in Non-Valvular AF Patients Treated With Factor-Xa Inhibitor, Dabigatran, or Warfarin After PSSW [file jamanetwopen-e2243307-s001.pdf]

Supplemental Online Content

Chan YH, Chao TF, Chen SW, et al. Development of interstitial lung disease among patients with atrial fibrillation receiving oral anticoagulants in Taiwan. *JAMA Netw Open*. 2022;5(11):e2243307. doi:10.1001/jamanetworkopen.2022.43307

eTable 1. International Classification of Disease (9th and 10th Edition) Clinical Modification (ICD 9-CM and ICD 10-CM) Codes Used to Define the Idiopathic Interstitial Lung Disease (ILD) in the Study Cohort

eTable 2. International Classification of Disease (9th and 10th Edition) Clinical Modification (ICD 9-CM and ICD 10-CM) Codes Used to Define the Co-Morbidities and Clinical Outcomes in the Study Cohort

eTable 3. Baseline Characteristics of Patients With Non-Valvular AF Treated With Factor-Xa Inhibitor, Dabigatran, or Warfarin After PSSW

eTable 4. The Incidence and Absolute Rate Difference of Interstitial Lung Disease (ILD), Thromboembolism, and Major Bleeding Among Patients With Non-Valvular AF Treated With Factor-Xa Inhibitor, Dabigatran, or Warfarin After PSSW

eTable 5. Baseline Characteristics of Non-Valvular AF Patients Treated With Factor-Xa Inhibitor, Dabigatran, or Warfarin Before PSSW: NOAC Without Prior Warfarin Exposure (At Least 180 Days Wash-Out Period)

eTable 6. Baseline Characteristics of Non-Valvular AF Patients Treated With Factor-Xa Inhibitor, Dabigatran, or Warfarin After PSSW: NOAC Without Prior Warfarin Exposure (At Least 180 Days Wash-Out Period)

eFigure 1. Graphical Depiction of Longitudinal Study Designs

eFigure 2. Subgroup Analysis of Hazard Ratio (HR) for Interstitial Lung Disease (ILD) for Patients Non-Valvular Atrial Fibrillation (AF) Treated With Factor-Xa Inhibitor and Warfarin After Propensity Score Stabilized Weights (PSSW)

eFigure 3. Subgroup Analysis of HR for ILD for Non-Valvular AF Patients Treated With Dabigatran and Warfarin After PSSW

© 2022 Chan YH et al. *JAMA Network Open.*

eFigure 4. The Impact of Amiodarone on the Risk of ILD in Non-Valvular AF Patients Treated With Factor-Xa Inhibitor, Dabigatran, or Warfarin After PSSW

This supplemental material has been provided by the authors to give readers additional information about their work.

© 2022 Chan YH et al. *JAMA Network Open.*

eTable 1. International Classification of Disease (9th and 10th edition) Clinical Modification (ICD 9-CM and ICD 10-CM) codes used to define the idiopathic interstitial lung disease (ILD) in the study cohort

ICD-9 Codes		ICD-10 Codes	
515	Post inflammatory pulmonary fibrosis	J84.10<br>J84.89	Pulmonary fibrosis, unspecified<br>Other specified interstitial pulmonary disease
516.0	Pulmonary alveolar proteinosis	J84.01	Alveolar proteinosis
516.1	Idiopathic pulmonary hemosiderosis	J84.03	Idiopathic pulmonary hemosiderosis
516.2	Pulmonary alveolar microlithiasis	J84.02	Pulmonary alveolar microlithiasis
516.30	Idiopathic interstitial pneumonia	J84.111	Idiopathic interstitial pneumonia
516.31	Idiopathic pulmonary fibrosis	J84.112	Idiopathic pulmonary fibrosis
516.32	Idiopathic non-specific interstitial pneumonitis	J84.113	Idiopathic non-specific interstitial pneumonitis
516.33	Acute interstitial pneumonitis	J84.114	Acute interstitial pneumonitis
516.34	Respiratory bronchiolitis inter lung dis	J84.115	Respiratory bronchiolitis inter lung dis
516.35	Idiopathic lymphoid inter. Pneumonia	J84.2	Idiopathic lymphoid inter. Pneumonia
516.36	Cryptogenic organizing pneumonia	J84.116	Cryptogenic organizing pneumonia
516.37	Desquamative interstitial pneumonia	J84.117	Desquamative interstitial pneumonia
516.4	Lymphangioleiomyomatosis	J84.81	Lymphangioleiomyomatosis
516.5	Adult pulmonary Langerhans cell histiocytosis	J84.82	Adult pulmonary Langerhans cell histiocytosis
516.61	Neuroendocrine cell hyperplasia of	J84.841	Neuroendocrine cell hyperplasia of

© 2022 Chan YH et al. *JAMA Network Open.*

	infancy		infancy
516.62	Pulmonary interstitial glycogenosis	J84.842	Pulmonary interstitial glycogenosis
516.63	Surfactant mutations of the lung	J84.83	Surfactant mutations of the lung
516.64	Alveolar cap dysplasia w/vein misalignment	J84.843	Alveolar cap dysplasia w/vein misalignment
516.69	Other interstitial lung disease of childhood	J84.848	Other interstitial lung disease of childhood
516.9	Other nonspecific alveolar & parietoalveolar pneumonpathies	J84.9	Interstitial pulmonary disease, unspecified

© 2022 Chan YH et al. *JAMA Network Open.*

eTable 2. International Classification of Disease (9th and 10th edition) Clinical Modification (ICD 9-CM and ICD 10-CM) codes used to define the co-morbidities and clinical outcomes in the study cohort

Disease	ICD-9 Codes	ICD-10 Codes	Diagnosis definition
Atrial fibrillation	427.31	I48	Discharge or outpatient department ≥2
Ischemic stroke	433, 434, 436	I63, I64	Discharge
Systemic embolism	444	I74	Discharge
Transient ischemic attack	435	G45	Discharge
Acute Myocardial infarction	410	I21-I23	Discharge
Ischemic heart disease	410, 411, 412, 413, 414	I21-I25	Outpatient department ≥2
Congestive heart failure	428	I11.0, I13.0, I13.2, I42.0, I50, I50.1, I50.9	Discharge
Hypertension	401, 402	I10-I16	Outpatient department ≥2
Diabetes mellitus	250	E11-E14	Outpatient department ≥2
Hyperlipidemia	272	E78	Outpatient department ≥2
Chronic gout	274.0, 274.10, 274.11, 274.19, 274.81, 274.82, 274.89, 274.9	M10, M1A	Outpatient department ≥2
Chronic lung disease	490, 491.0, 491.1, 491.20-491.22, 491.8, 491.9, 492.0, 492.8, 493.00-493.02 493.10-493.12, 493.20-493.22, 493.81, 493.82, 493.90-493.92, 494.0, 494.1, 495.8, 495.9,	J41-J44	Outpatient department ≥2

© 2022 Chan YH et al. *JAMA Network Open.*

	496, 500, 502, 503, 504, 505, A323, A325		
Chronic kidney disease	580-589	I12, I13, N00, N01, N02, N03, N04, N05, N07, N11, N14, N17, N18, N19, Q61	Outpatient department ≥2
Chronic liver disease	570, 571, 572	B150, B160, B162, B190, K704, K72, K766, I85	Outpatient department ≥2
Malignancy	140.0-208.9	C	Outpatient department ≥2

© 2022 Chan YH et al. *JAMA Network Open.*

eTable 3. Baseline characteristics of patients with non-valvular AF treated with factor-Xa inhibitor, dabigatran, or warfarin after PSSW

	Factor Xa inhibitor (n = 64,393.72)	Dabigatran (n = 22,178.67)	Warfarin (n = 18,469.65)	ASMD: Factor Xa inhibitor vs. Warfarin	ASMD: Dabigatran vs. Warfarin
**Age**					
**(mean ± STD)**	73.6±11.6	73.5±11.4	73.3±12.2	0.0265	0.0195
**<65**	14318.70 (22.24%)	4890.67 (22.05%)	4160.75 (22.53%)	0.0282	0.0282
**65-74**	18435.32 (28.63%)	6372.68 (28.73%)	5143.34 (27.85%)		
**75-84**	20559.84 (31.93%)	7103.54 (32.03%)	5905.56 (31.97%)		
**>85**	11079.86 (17.21%)	3811.78 (17.19%)	3260.00 (17.65%)		
**Male**	36421.26 (56.56%)	12599.74 (56.81%)	10398.16 (56.30%)	0.0053	0.0104
**CHA_2DS_2-VASc (mean ± STD)**	3.2±1.7	3.2±1.7	3.2±1.7	0.0038	0.0037
**HAS-BLED (mean ± STD)**	2.5±1.2	2.5±1.2	2.5±1.2	0.0336	0.0285
**Hypertension**	32361.67 (50.26%)	11071.96 (49.92%)	9214.61 (49.89%)	0.0074	0.0006
**Diabetes mellitus**	22339.79 (34.69%)	7703.44 (34.73%)	6430.42 (34.82%)	0.0026	0.0018
**Dyslipidemia**	27326.59 (42.44%)	9346.67 (42.14%)	7687.20 (41.62%)	0.0167	0.0107
**Chronic live disease**	5333.76 (8.28%)	1820.47 (8.21%)	1506.09 (8.15%)	0.0047	0.0020
**Chronic kidney disease**	10034.06 (15.58%)	3407.56 (15.36%)	2936.50 (15.90%)	0.0088	0.0149
**Gout**	9672.22 (15.02%)	3290.48 (14.84%)	2797.60 (15.15%)	0.0036	0.0088
**Congestive heart failure**	5566.95 (8.65%)	1873.72 (8.45%)	1676.50 (9.08%)	0.0153	0.0225
**Chronic ischemic heart disease**	6651.41 (10.33%)	2271.33 (10.24%)	1900.64 (10.29%)	0.0013	0.0016
**Stroke**	11992.68 (18.62%)	4116.23 (18.56%)	3399.75 (18.41%)	0.0056	0.0040
**Cancer**	6335.71 (9.84%)	2142.37 (9.66%)	1825.70 (9.88%)	0.0015	0.0077
**Rheumatoid arthritis**	209.77 (0.33%)	65.86 (0.30%)	47.37 (0.26%)	0.0129	0.0078
**PCI**	4146.51 (6.44%)	1401.84 (6.32%)	1173.05 (6.35%)	0.0036	0.0013
**CABG**	365.05 (0.57%)	103.56 (0.47%)	102.00 (0.55%)	0.0020	0.0121
**History of bleeding**	991.11 (1.54%)	328.14 (1.48%)	300.86 (1.63%)	0.0072	0.0122
**Use of NSAIDs**	15909.02 (24.71%)	5460.48 (24.62%)	4548.21 (24.63%)	0.0019	0.0001
**Use of PPI**	7653.56 (11.89%)	2584.31 (11.65%)	2197.10 (11.90%)	0.0003	0.0076
**Use of H_2 blocker**	20365.21 (31.63%)	7044.47 (31.76%)	5931.67 (32.12%)	0.0106	0.0077
**Use of ACEI or ARB**	37938.29 (58.92%)	13069.26 (58.93%)	10936.28 (59.21%)	0.0061	0.0059
**Use of beta-blocker**	38981.94 (60.54%)	13392.62 (60.39%)	11195.55 (60.62%)	0.0016	0.0048
**Use of verapamil or diltiazem**	14689.91 (22.81%)	5005.83 (22.57%)	4282.69 (23.19%)	0.0090	0.0148
**Use of statin**	21675.78 (33.66%)	7433.07 (33.51%)	6037.39 (32.69%)	0.0208	0.0177
**Use of antiplatelet**	34701.04 (53.89%)	12004.07 (54.12%)	10132.04 (54.86%)	0.0196	0.0149
**Use of amiodarone**	20276.05 (31.49%)	6910.87 (31.16%)	5911.67 (32.01%)	0.0113	0.0184
**Use of dronedarone**	2032.89 (3.16%)	656.91 (2.96%)	560.31 (3.03%)	0.0072	0.0043
**Use of chemotherapy**	913.69 (1.42%)	291.56 (1.31%)	257.77 (1.40%)	0.0020	0.0071
**Use of target therapy**	822.63 (1.28%)	260.13 (1.17%)	219.26 (1.19%)	0.0082	0.0013
**Use of methotrexate**	174.96 (0.27%)	52.40 (0.24%)	45.92 (0.25%)	0.0046	0.0025
**Use of anti-TNF agent**	34.71 (0.05%)	12.13 (0.05%)	6.53 (0.04%)	0.0088	0.0092
**Use of steroid**	1669.95 (2.59%)	542.90 (2.45%)	497.37 (2.69%)	0.0063	0.0156

© 2022 Chan YH et al. *JAMA Network Open.*

**Use of quinidine**	73.58 (0.11%)	22.60 (0.10%)	18.90 (0.10%)	0.0037	0.0001
**Use of rifampicin**	191.63 (0.30%)	59.22 (0.27%)	54.59 (0.30%)	0.0004	0.0054
**Use of macrolides**	1428.78 (2.22%)	466.90 (2.11%)	420.71 (2.28%)	0.0040	0.0119
**Use of anti-fungal agent**	565.49 (0.88%)	184.01 (0.83%)	168.97 (0.91%)	0.0039	0.0093

ACEI = angiotensin-converting-enzyme inhibitor; AF = atrial fibrillation; APT = antiplatelet agent; ARB = angiotensin II receptor antagonists; ASMD = absolute standardized mean difference; CABG = coronary artery bypass grafting; CHA_2DS_2-VASc = congestive heart failure, hypertension, age 75 years or older, diabetes mellitus, previous stroke/transient ischemic attack, vascular disease, age 65 to 74 years, female; HAS-BLED = hypertension, abnormal renal or liver function, stroke, bleeding history, labile INR, age 65 years or older, and antiplatelet drug or alcohol use; NOAC = non-vitamin K antagonist oral anticoagulant; NSAIDs = non-steroidal anti-inflammatory drugs; OACs = oral anticoagulants; PCI = Percutaneous coronary intervention; PPI = proton pump inhibitor; PSSW = propensity score stabilized weighting; STD = standard deviation; TNF = tumor necrosis factor

© 2022 Chan YH et al. *JAMA Network Open.*

eTable 4. The incidence and absolute rate difference of interstitial lung disease (ILD), thromboembolism, and major bleeding among patients with non-valvular AF treated with factor-Xa inhibitor, dabigatran, or warfarin after PSSW

	**Incidence (100pt per yr) (95%CI)**			**Rate difference (100pt per yr) (95%CI)**	
	**Factor Xa inhibitor (n = 64,393.72)**	**Dabigatran (n = 22,178.67)**	**Warfarin (n = 18,469.65)**	**Factor Xa inhibitor**	**Dabigatran**
				**vs.**	**vs.**
				**Warfarin**	**Warfarin**
**Outcomes**					
**ILD**	0.29 (0.26-0.32)	0.22 (0.18-0.26)	0.17 (0.13-0.21)	0.12 (0.08, 0.17)	0.05 (-0.001, 0.10)
**IS/SE**	1.76 (1.69-1.84)	1.91 (1.79-2.02)	2.55 (2.41-2.69)	-0.78 (-0.94, -0.63)	-0.64 (-0.82, -0.46)
**ICH**	0.46 (0.42-0.49)	0.35 (0.30-0.40)	0.90 (0.81-0.98)	-0.44 (-0.53, -0.35)	-0.54 (-0.64, -0.45)
**Major GI bleeding**	1.04 (0.99-1.09)	0.94 (0.86-1.02)	1.32 (1.22-1.42)	-0.28 (-0.40, -0.17)	-0.38 (-0.51, -0.26)
**All major bleeding**	1.61 (1.54-1.68)	1.38 (1.28-1.47)	2.39 (2.25-2.52)	-0.78 (-0.93, -0.63)	-1.01 (-1.17, -0.84)

GI = gastrointestinal bleeding; ICH = intracranial hemorrhage; ILD = interstitial lung disease; IS/SE = ischemic stroke/systemic embolism,

© 2022 Chan YH et al. *JAMA Network Open.*

eTable 5. Baseline characteristics of non-valvular AF patients treated with factor Xa inhibitor, dabigatran, or warfarin before PSSW: NOAC without prior warfarin exposure (at least 180 days wash-out period)

	Factor Xa inhibitor	Dabigatran	Warfarin	ASMD	
				Factor Xa inhibitor vs.	Dabigatran vs.
	(n = 61,713)	(n = 21,035)	(n = 18,988)	Warfarin	Warfarin
**Age**					
**(mean ± STD)**	75.0±11.1	73.4±10.8	69.4±14.1	0.4226	0.3193
**<65**	10880 (17.63%)	4164 (19.80%)	7536 (39.69%)	0.5009	0.4693
**65-74**	18247 (29.57%)	6889 (32.75%)	3906 (20.57%)		
**75-84**	20801 (33.71%)	6968 (33.13%)	4715 (24.83%)		
**>85**	11785 (19.10%)	3014 (14.33%)	2831 (14.91%)		
**Male**	34068 (55.20%)	12721 (60.48%)	10760 (56.67%)	0.0295	0.0774
**CHA_2DS_2-VASc (mean ± STD)**	3.3±1.7	3.2±1.6	2.6±1.9	0.3782	0.3081
**HAS-BLED (mean ± STD)**	2.6±1.2	2.5±1.1	2.1±1.3	0.4004	0.3405
**Hypertension**	32930 (53.36%)	10240 (48.68%)	7904 (41.63%)	0.2366	0.1421
**Diabetes mellitus**	22362 (36.24%)	7291 (34.66%)	5702 (30.03%)	0.1321	0.0991
**Dyslipidemia**	28121 (45.57%)	8676 (41.25%)	6411 (33.76%)	0.2431	0.1550
**Chronic live disease**	5304 (8.59%)	1664 (7.91%)	1485 (7.82%)	0.0282	0.0033
**Chronic kidney disease**	10758 (17.43%)	2499 (11.88%)	2600 (13.69%)	0.1033	0.0543
**Gout**	9808 (15.89%)	2909 (13.83%)	2590 (13.64%)	0.0635	0.0055
**Congestive heart failure**	5431 (8.80%)	1487 (7.07%)	1769 (9.32%)	0.0180	0.0820
**Chronic ischemic heart disease**	7014 (11.37%)	1880 (8.94%)	1650 (8.69%)	0.0892	0.0087
**Stroke**	11273 (18.27%)	4855 (23.08%)	2459 (12.95%)	0.1469	0.2659
**Cancer**	6545 (10.61%)	1811 (8.61%)	1665 (8.77%)	0.0621	0.0057
**Rheumatoid arthritis**	217 (0.35%)	67 (0.32%)	49 (0.26%)	0.0170	0.0113
**PCI**	4522 (7.33%)	1095 (5.21%)	955 (5.03%)	0.0956	0.0080
**CABG**	339 (0.55%)	49 (0.23%)	210 (1.11%)	0.0615	0.1072
**History of bleeding**	973 (1.58%)	275 (1.31%)	305 (1.61%)	0.0024	0.0250
**Use of NSAIDs**	15242 (24.70%)	5001 (23.77%)	4866 (25.63%)	0.0214	0.0430
**Use of PPI**	7564 (12.26%)	1906 (9.06%)	2551 (13.43%)	0.0352	0.1388
**Use of H_2 blocker**	19306 (31.28%)	6632 (31.53%)	6132 (32.29%)	0.0217	0.0164
**Use of ACEI or ARB**	36753 (59.55%)	12392 (58.91%)	10539 (55.50%)	0.0820	0.0689
**Use of beta-blocker**	37467 (60.71%)	12062 (57.34%)	11739 (61.82%)	0.0228	0.0914
**Use of verapamil or diltiazem**	13972 (22.64%)	4370 (20.77%)	4685 (24.67%)	0.0479	0.0931
**Use of statin**	22080 (35.78%)	7232 (34.38%)	4835 (25.46%)	0.2252	0.1957
**Use of antiplatelet**	33097 (53.63%)	11339 (53.91%)	10455 (55.06%)	0.0287	0.0232
**Use of amiodarone**	18670 (30.25%)	5135 (24.41%)	8065 (42.47%)	0.2561	0.3901
**Use of dronedarone**	2544 (4.12%)	315 (1.50%)	322 (1.70%)	0.1448	0.0158
**Use of chemotherapy**	947 (1.53%)	242 (1.15%)	263 (1.39%)	0.0125	0.0210
**Use of target therapy**	971 (1.57%)	208 (0.99%)	141 (0.74%)	0.0777	0.0266
**Use of methotrexate**	172 (0.28%)	51 (0.24%)	51 (0.27%)	0.0019	0.0052
**Use of anti-TNF agent**	35 (0.06%)	14 (0.07%)	11 (0.06%)	0.0005	0.0035
**Use of steroid**	1631 (2.64%)	428 (2.03%)	583 (3.07%)	0.0257	0.0657

© 2022 Chan YH et al. *JAMA Network Open.*

**Use of quinidine**	67 (0.11%)	22 (0.10%)	49 (0.26%)	0.0350	0.0361
**Use of rifampicin**	184 (0.30%)	50 (0.24%)	74 (0.39%)	0.0156	0.0272
**Use of macrolides**	1368 (2.22%)	358 (1.70%)	504 (2.65%)	0.0284	0.0653
**Use of anti-fungal agent**	546 (0.88%)	109 (0.52%)	261 (1.37%)	0.0464	0.0885

The abbreviations as in **eTable 3**

© 2022 Chan YH et al. *JAMA Network Open.*

eTable 6. Baseline characteristics of non-valvular AF patients treated with factor Xa inhibitor, dabigatran, or warfarin after PSSW: NOAC without prior warfarin exposure (at least 180 days wash-out period)

	**Factor Xa inhibitor**	**Dabigatran**	**Warfarin**	**ASMD**	
				**Factor Xa inhibitor**	**Dabigatran**
				**vs.**	**vs.**
	**(n = 61,547.15)**	**(n = 20,712.68)**	**(n = 18,488.98)**	**Warfarin**	**Warfarin**
**Age**					
**(mean ± STD)**	73.7±11.6	73.6±11.3	73.4±12.2	0.0272	0.0198
**<65**	13601.00 (22.10%)	4540.73 (21.92%)	4140.94 (22.40%)	0.0304	0.0304
**65-74**	17601.86 (28.60%)	5952.45 (28.74%)	5136.28 (27.78%)		
**75-84**	19668.98 (31.96%)	6630.25 (32.01%)	5917.45 (32.01%)		
**>85**	10675.31 (17.34%)	3589.25 (17.33%)	3294.31 (17.82%)		
**Male**	34806.13 (56.55%)	11764.15 (56.80%)	10397.52 (56.24%)	0.0064	0.0114
**CHA_2DS_2-VASc (mean ± STD)**	3.2±1.7	3.2±1.7	3.2±1.7	0.0065	0.0051
**HAS-BLED (mean ± STD)**	2.5±1.2	2.5±1.2	2.5±1.2	0.0324	0.0283
**Hypertension**	30945.94 (50.28%)	10372.58 (50.08%)	9228.00 (49.91%)	0.0074	0.0034
**Diabetes mellitus**	21381.58 (34.74%)	7226.62 (34.89%)	6454.11 (34.91%)	0.0035	0.0004
**Dyslipidemia**	26166.48 (42.51%)	8752.09 (42.25%)	7713.72 (41.72%)	0.0162	0.0109
**Chronic live disease**	5089.78 (8.27%)	1709.64 (8.25%)	1504.60 (8.14%)	0.0048	0.0043
**Chronic kidney disease**	9616.43 (15.62%)	3195.95 (15.43%)	2941.71 (15.91%)	0.0079	0.0134
**Gout**	9261.90 (15.05%)	3084.22 (14.89%)	2806.72 (15.18%)	0.0037	0.0082
**Congestive heart failure**	5272.90 (8.57%)	1726.05 (8.33%)	1660.01 (8.98%)	0.0146	0.0232
**Chronic ischemic heart disease**	6392.83 (10.39%)	2140.72 (10.34%)	1912.10 (10.34%)	0.0015	0.0002
**Stroke**	11248.67 (18.28%)	3784.10 (18.27%)	3371.09 (18.23%)	0.0011	0.0010
**Cancer**	6073.38 (9.87%)	2004.34 (9.68%)	1827.37 (9.88%)	0.0005	0.0070
**Rheumatoid arthritis**	199.93 (0.32%)	62.76 (0.30%)	47.02 (0.25%)	0.0132	0.0093
**PCI**	3984.10 (6.47%)	1312.70 (6.34%)	1180.01 (6.38%)	0.0037	0.0018
**CABG**	345.87 (0.56%)	92.18 (0.45%)	103.31 (0.56%)	0.0004	0.0163
**History of bleeding**	934.73 (1.52%)	300.36 (1.45%)	298.78 (1.62%)	0.0079	0.0136
**Use of NSAIDs**	15214.17 (24.72%)	5103.09 (24.64%)	4552.48 (24.62%)	0.0023	0.0003
**Use of PPI**	7269.16 (11.81%)	2390.93 (11.54%)	2184.28 (11.81%)	0.0001	0.0085
**Use of H_2 blocker**	19373.43 (31.48%)	6541.75 (31.58%)	5903.99 (31.93%)	0.0099	0.0076
**Use of ACEI or ARB**	36159.86 (58.75%)	12171.73 (58.76%)	10925.61 (59.09%)	0.0070	0.0067
**Use of beta-blocker**	37097.94 (60.28%)	12441.66 (60.07%)	11157.56 (60.35%)	0.0015	0.0058
**Use of verapamil or diltiazem**	13944.79 (22.66%)	4637.08 (22.39%)	4249.24 (22.98%)	0.0078	0.0144
**Use of statin**	20685.87 (33.61%)	6950.54 (33.56%)	6025.48 (32.59%)	0.0218	0.0208
**Use of antiplatelet**	33190.01 (53.93%)	11210.68 (54.12%)	10144.85 (54.87%)	0.0191	0.0151
**Use of amiodarone**	19255.55 (31.29%)	6411.56 (30.95%)	5866.32 (31.73%)	0.0096	0.0169
**Use of dronedarone**	1933.58 (3.14%)	607.70 (2.93%)	556.29 (3.01%)	0.0078	0.0045
**Use of chemotherapy**	877.21 (1.43%)	275.21 (1.33%)	253.17 (1.37%)	0.0048	0.0036
**Use of target therapy**	798.81 (1.30%)	247.85 (1.20%)	222.82 (1.21%)	0.0084	0.0008
**Use of methotrexate (MTX)**	164.76 (0.27%)	49.06 (0.24%)	43.91 (0.24%)	0.0061	0.0001
**Use of anti-TNF agent**	32.03 (0.05%)	10.64 (0.05%)	6.15 (0.03%)	0.0091	0.0089
**Use of steroid**	1590.62 (2.58%)	500.31 (2.42%)	495.81 (2.68%)	0.0061	0.0171

© 2022 Chan YH et al. *JAMA Network Open.*

**Use of quinidine**	70.41 (0.11%)	21.34 (0.10%)	18.76 (0.10%)	0.0040	0.0005
**Use of rifampicin**	181.91 (0.30%)	53.46 (0.26%)	54.11 (0.29%)	0.0005	0.0067
**Use of macrolides**	1355.76 (2.20%)	429.79 (2.08%)	419.19 (2.27%)	0.0044	0.0133
**Use of anti-fungal agent**	543.86 (0.88%)	168.48 (0.81%)	170.51 (0.92%)	0.0041	0.0119

The abbreviations as in **eTable 3**

© 2022 Chan YH et al. *JAMA Network Open.*

eFigure 1. Graphical depiction of longitudinal study designs

From June 1, 2012, to December 31, 2017, a total of 122,345 atrial fibrillation (AF) patients with the first prescription of factor Xa inhibitor, dabigatran, or warfarin between 2012/06/01 to 2017/12/31 were identified. There were 113,239 non-valvular AF patients treated with oral anticoagulants (OACs) after excluding the diagnosis indicating venous thromboembolism, post valvular surgery, mitral stenosis, or end-stage renal disease at baseline before drug-index date. After excluding 7,195 non-valvular AF patients with a diagnosis of any chronic lung disease before the drug-index date, a total of 64,555, 22,501, and 18,988 non-valvular AF patients without a prior diagnosis of chronic lung disease treated with factor Xa inhibitor, dabigatran, and warfarin, respectively, were enrolled. The index date was defined as the first prescription date for the index drug. The follow-up period was defined as the duration from the index date until the first occurrence of study outcome (ILD), death, or until the end date of the study period (December 31, i2019), whichever came first.

Abbreviations: AF = atrial fibrillation; ILD = interstitial lung disease; OAC = oral anticoagulant

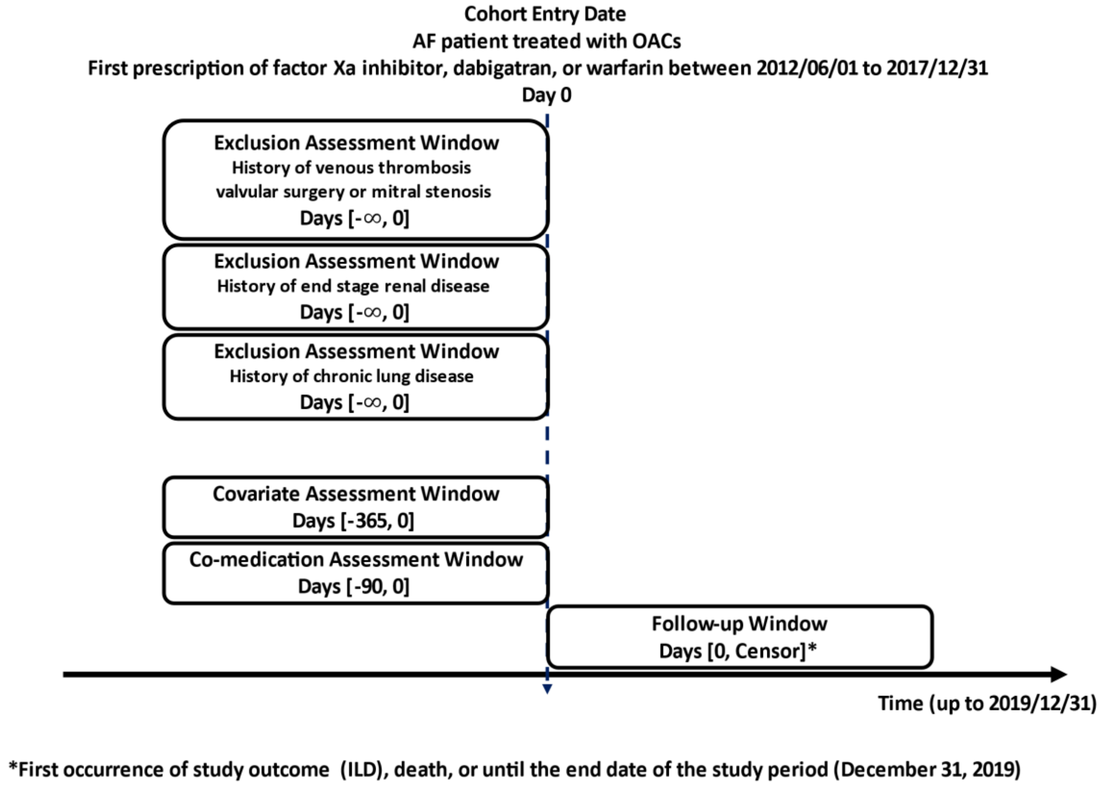

© 2022 Chan YH et al. *JAMA Network Open.*

eFigure 2. Subgroup analysis of hazard ratio (HR) for interstitial lung disease (ILD) for patients non-valvular atrial fibrillation (AF) treated with factor Xa inhibitor and warfarin after propensity score stabilized weights (PSSW)

Subgroup analysis showed that three factor Xa inhibitors were all associated with a higher risk of incident ILD when compared to warfarin after PSSW. Subgroup analysis shows that a higher risk of incident ILD for factor Xa inhibitor over warfarin was consistent irrespective of age, sex, use of angiotensin system inhibitor, amiodarone, statin, or beta-blocker, risk of stroke or major bleeding (*P* interaction all > 0.05)

Abbreviations: ACEI = angiotensin-converting-enzyme inhibitor; AF = atrial fibrillation; ARB = angiotensin II receptor antagonists; CHA_2DS_2-VASc = congestive heart failure, hypertension, age 75 years or older, diabetes mellitus, previous stroke/transient ischemic attack, vascular disease, age 65 to 74 years, female; CI = confidential interval; HAS-BLED = hypertension, abnormal renal or liver function, stroke, bleeding history, labile INR, age 65 years or older, and antiplatelet drug or alcohol use; HR = hazard ratio; ILD = interstitial lung disease; PSSW = propensity score stabilized weighting

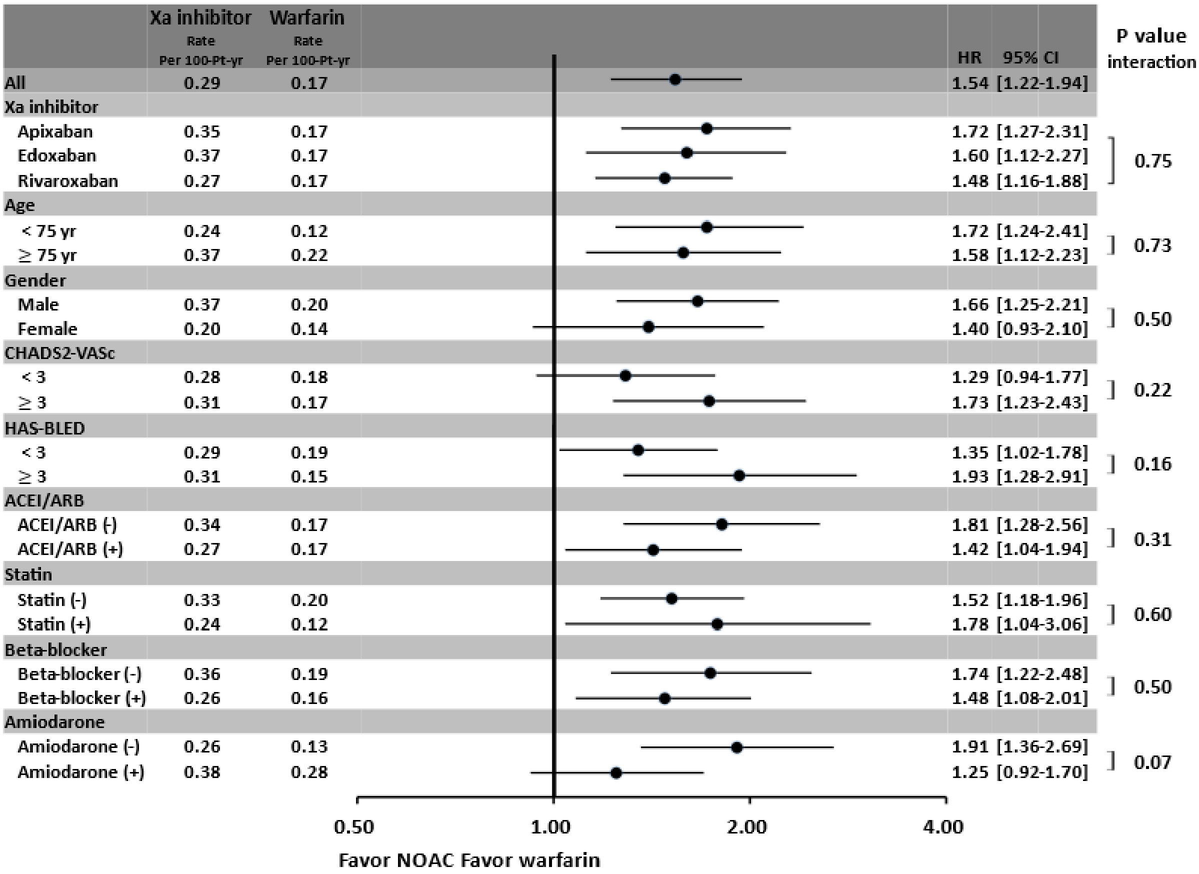

© 2022 Chan YH et al. *JAMA Network Open.*

eFigure 3. Subgroup analysis of HR for ILD for non-valvular AF patients treated with dabigatran and warfarin after PSSW

Use of dabigatran was not associated with a risk of incident ILD persisted in all subgroups compared to warfarin (*P* interaction all > 0.05).

The abbreviations as in **eFigure 2**

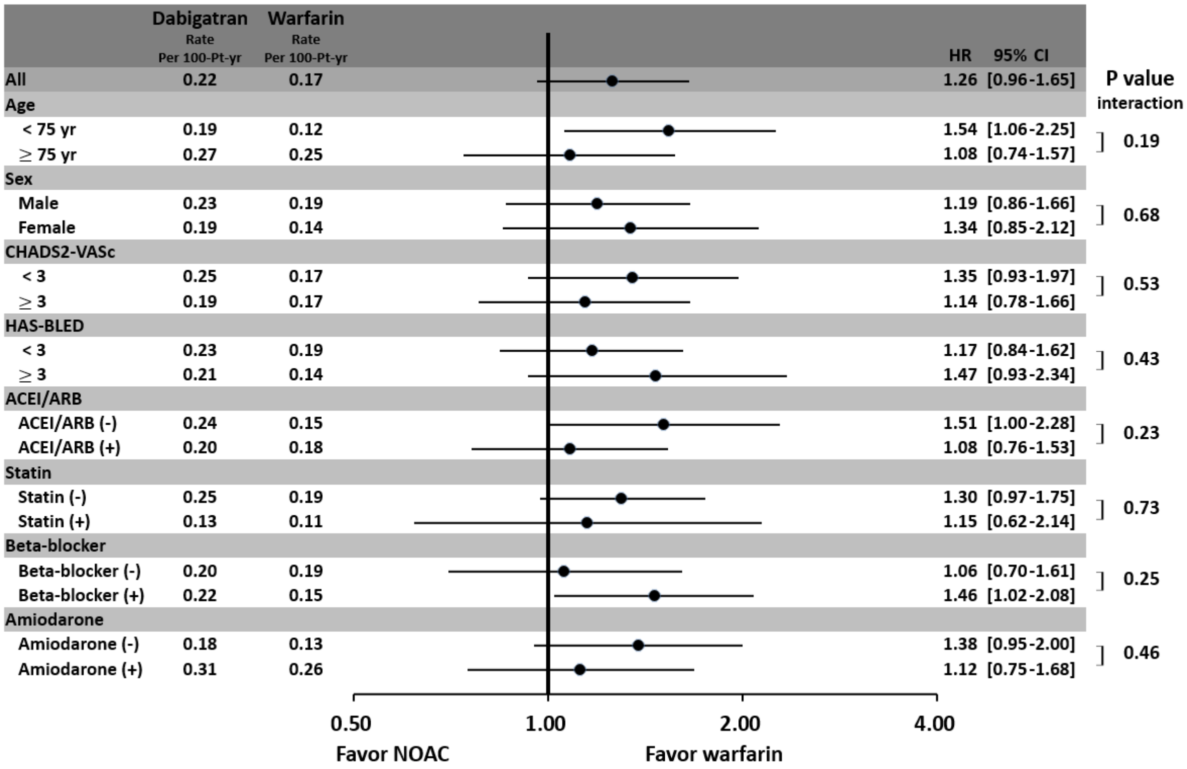

© 2022 Chan YH et al. *JAMA Network Open.*

eFigure 4. The impact of amiodarone on the risk of ILD in non-valvular AF patients treated with factor Xa inhibitor, dabigatran, and warfarin after PSSW

Amiodarone, expected to be frequently co-prescribed with NOACs in patients with AF, was prescribed in 30.7%, 24.7%, and 42.5% of patients treated with factor Xa inhibitors, dabigatran, and warfarin in the present study. The use of amiodarone was strongly associated with a higher risk of incident ILD in patients treated with either factor-Xa inhibitor, dabigatran, or warfarin. Of note, those patients co-treated with amiodarone and factor-Xa inhibitors were associated with the highest risk of incident ILD, whereas those treated with warfarin but without amiodarone were associated with the lowest risk of incident ILD.

The abbreviations as in **eFigure 2**

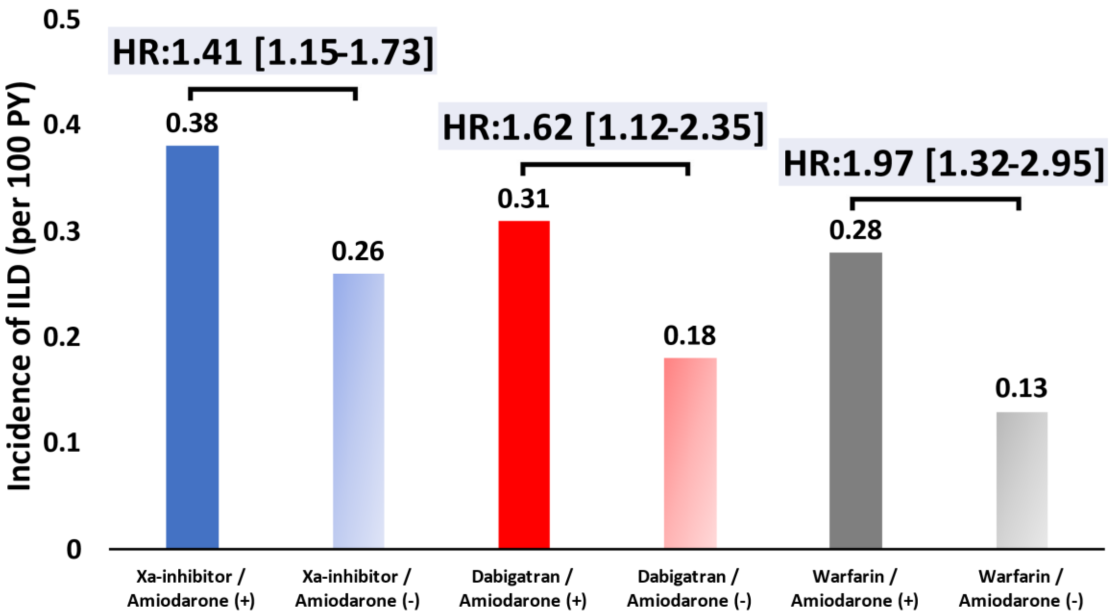

© 2022 Chan YH et al. *JAMA Network Open.*
